# *Selenobaculum gbiensis* gen. nov. sp. nov., a new bacterium isolated from the gut microbiota of a patient with Crohn’s disease

**DOI:** 10.1038/s41598-023-42017-0

**Published:** 2023-09-08

**Authors:** Soyoung Yeo, Hyunjoon Park, Heebal Kim, Chang Beom Ryu, Chul Sung Huh

**Affiliations:** 1 Department of Agricultural Biotechnology, College of Agriculture and Life Sciences, Seoul National University, Seoul, 08826 South Korea; 2 Research Institute of Eco-Friendly Livestock Science, Institute of Green-Bio Science and Technology, Seoul National University, Pyeongchang, 25354 South Korea; 3 Department of Internal Medicine, Digestive Disease Center and Research Institute, Soon Chun Hyang University School of Medicine, Bucheon, 14584 South Korea; 4 Graduate School of International Agricultural Technology, Seoul National University, Pyeongchang, 25354 South Korea

**Keywords:** Bacteria, Clinical microbiology

## Abstract

The human gut microbiota is a complex ecology comprising approximately 10 to 100 trillion microbial cells. Most of the bacteria detected by 16s rRNA sequencing have yet to be cultured, but intensive attempts to isolate the novel bacteria have improved our knowledge of the gut microbiome composition and its roles within human host. In our culturomics study, a novel gram-negative, motile, obligately anaerobic, rod-shaped bacteria, designated as strain ICN-92133^T^, was isolated from a fecal sample of a 26-year-old patient with Crohn’s disease. Based on the 16s rRNA sequence of strain ICN-92133^T^, the phylogeny analysis placed the strain into the family *Selenomonadaceae*, showing 93.91% similarity with the closely related *Massilibacillus massiliensis* strain DSM 102838^T^. Strain ICN-92133^T^ exhibited a genome size of 2,679,003 bp with a GC content of 35.5% which was predicted to contain 26 potential virulence factors and five antimicrobial resistance genes. In comparative genomic analysis, strain ICN-92133^T^ showed digital DNA–DNA Hybridization and OrthoANI values lower than 21.9% and 71.9% with the closest type strains, respectively. In addition, comparing phenotypic, biochemical, and cellular fatty acids with those of closely related strains revealed the distinctiveness of strain ICN-92133^T^. Based on the taxonogenomic results, strain ICN-92133^T^ is proposed as a novel species belonging to a new genus. Therefore, we suggest the name of the new genus *Selenobaculum* gen. nov. within the family *Selenomonadaceae* and strain ICN-92133^T^ (= KCTC 25622^T^ = JCM 36070^T^) as a type strain of new species *Selenobaculum gbiensis* sp. nov.

## Introduction

The gut microbiota is linked to human health and disease by maintaining intestinal homeostasis and regulating metabolism^1–3^. Since the first large-scale 16s rRNA sequencing in 2005 to identify intestinal bacteria, information on the diversity of gut ecology has increased exponentially^4,5^. However, the culture of bacteria had been very biased toward partial phylogenetic groups, and the quantitative gap between the number of novel cultured species and the results obtained by the culture-independent method had widened^6,7^. Microbial culturomics, introduced in 2012, is a research concept that combines high-throughput cultivating microorganisms with various culture conditions, a matrix-assisted laser desorption-ionization time-of-flight mass spectrometry (MALDI-TOF MS), and 16s rRNA gene sequencing^8^. The culturomics approach has enabled high-throughput microbial identification and cultivation, resulting in many previously unexplored bacterial lineages reported^9–11^.

Nevertheless, a significant fraction of the bacteria detected in the 16s rRNA sequencing has yet to be cultured. The number is expected to be higher when considering the bacteria not detected in metagenomics for reasons such as low abundances and sequencing bias^12,13^. Since 2021, we have conducted a culturomics study focusing on the intestine of healthy individuals and inflammatory bowel disease (IBD) patients to contribute to discovering novel gut bacteria. As part of the results, we isolated a novel bacterial stain ICN-92133^T^ (= KCTC 25622^T^ = JCM 36070^T^) from a stool sample in a patient with Crohn's disease. Using the taxonogenomics, a new research concept that proposes the novelty of new species based on whole genome sequence along with polyphasic properties^14^, we present distinct properties of strain ICN-92133^T^ from its phylogenetic neighbors, thereby proposing strain ICN-92133^T^ as a type species *Selenobaculum gbiensis* gen. nov., sp. nov. of the new genus within the family *Selenomonadaceae*.

## Materials and methods

### Sample collection under ethical approval

Before the sample collection, this study was approved by the Institutional Review Board of Soon Chun Hyang University Bucheon Hospital in South Korea under the number SCHBC 2021-01-028-002. The protocol was carried out in accordance with relevant guidelines and regulations approved by our ethics committee. The donor was a 26-year-old male diagnosed with Crohn’s disease who gave his informed and signed consent. The fecal samples were kept below 4 °C under anaerobic conditions immediately after defecation until processed in the laboratory within 24 h.

### Strain isolation and identification by MALDI-TOF MS

The sample was homogenized, resuspended to 0.25 g/L with 0.85% sodium chloride saline, and immobilized on polysaccharide gel beads for long-term pre-incubation^15^. The stool gel beads were inoculated in an anaerobic culture bottle (BioMérieux, Marcy l’Etoile, France) supplemented with 0.2 μm filtered rumen fluid^16^ and defibrinated sheep blood and then incubated at 37 °C for 30 days. The cultured broth was sampled regularly and plated onto modified Gifu Anaerobic Medium (mGAM; Nissui Pharmaceutical, Tokyo, Japan). Bacterial colonies were isolated and initially identified using MALDI-TOF MS on a Biotyper Sirius system (Bruker Daltonics, Bremen, Germany)^17^. The process was conducted in an anaerobic chamber consisting of 5% CO_2_, 10% H_2_, and 85% N_2_.

### Whole genome sequencing and analysis

Genomic DNA of strain ICN-92133^T^ was extracted using Quick-DNA HMW MagBead kit (Zymo Research Corporation, Irvine, CA, USA) with lysozyme chloride (Sigma-Aldrich, St. Louis, MO, USA) and quantified by QuantiFluor dsDNA System method (Promega, Madison, WI, USA) using Victor Nivo Multimode Microplate Reader (PerkinElmer, Waltham, MA, USA). Whole genome sequencing was performed using a PacBio Sequel (Pacific Biosciences, Menlo Park, CA, USA) and a NovaSeq 6000 system (Illumina, San Diego, CA, USA) by Macrogen Corporation (Seoul, South Korea). PacBio long-read sequences from approximately 12 kb SMRTbell templates were assembled de novo to construct genome sequences. They were then corrected using Illumina sequencing data from paired-end (2 × 150 bp) sequencing to obtain high quality. In this study, Microbial Assembly Application was applied for assembly^18^, and the genome was annotated using the PATRIC RAST tool kit (RASTtk)^19^ in the BV-BRC server^20^. The circular genome was constructed in a CGView tool^21^. The virulence factor database (VFDB)^22^ and the Comprehensive Antibiotic Resistance Database (CARD)^23^ were used to predict potential virulence factors and antimicrobial resistance genes.

### Phylogenetic analysis based on 16s rRNA gene sequences

The NCBI BLASTn search^24^ and Eztaxon database^25^ were used to classify the strain based on 16s rRNA gene sequence similarities with phylogenetically closest strains. The threshold of 94.5% 16s rRNA sequence identity, previously established^26,27^, was applied to determine a new genus classification. A phylogenetic tree was constructed using the Maximum-likelihood method^28^ based on the Tamura-Nei model^29^ after alignment of the 16s rRNA sequences using MUSCLE^30^ in the MEGA11 program^31^.

### Morphological, phenotypical, and biochemical assays

Gram-stained morphology was observed using a phase contrast microscope (Eclipse Ci-L, Nikon, Tokyo, Japan). The outer and inner structures of strain ICN-92133^T^ were observed using the scanning electron microscope (SEM) and energy-filtered transmission electron microscope (EFTEM) acquired on a Regulus 8100 (Hitachi, Tokyo, Japan) and LIBRA 120 (Carl Zeiss, Germany), respectively. The main characteristics, such as oxidase, catalase, nitrate reduction, and sporulation activities, of strain ICN-92133^T^ and phylogenetically closest type strains were compared. The optimal growth conditions were determined by culturing at different temperatures (20, 26, 30, 37, and 45 °C), pH (5, 5.5, 6, 6.5, 7, 7.5, 8.5), and osmotic conditions (0, 1, 3, 5, 10, 15 and 20 g of NaCl/L). According to the manufacturer's instructions, enzymatic activities and substrate utilization properties were investigated using API ZYM, API 20A, and API 50CH kits (BioMérieux) at 30 °C.

### Cellular fatty acids methyl ester analysis

Cellular fatty acids methyl esters (FAMEs) were extracted from colonies grown on mGAM agar and analyzed by Agilent 8890 gas chromatography system (Agilent Technologies, Inc., Palo Alto, CA, USA) at Korean Collection for Type Cultures (KCTC; Jeonbuk, South Korea). The spectra of cellular fatty acids were determined using the Sherlock MIDI software version 6.5 based on the ANAER6 method^32^.

### Minimum inhibitory concentration assay

The minimum inhibitory concentrations (MICs) of strain ICN-92133^T^ were determined in mGAM agar at 30 °C using ETEST (BioMérieux) of nine antibiotics: ampicillin, gentamicin, kanamycin, streptomycin, erythromycin, clindamycin, vancomycin, tetracycline, and chloramphenicol.

### Genomic comparison

Whole genome-based phylogeny was inferred by cross-genus protein families (PGfams) Codon Tree pipeline^33^ and Type (Strain) Genome Server (TYGS)^34^. The genomic features of the comparing strains were obtained from the BV-BRC server^20^. To calculate in silico genome-to-genome similarity, digital DNA-DNA hybridization (dDDH)^35^ and Orthologous Average Nucleotide Identity (OrthoANI)^36^ were applied.

## Results

### Strain isolation, morphology, and identification of strain ICN-92133^T^

**Figure 1:**
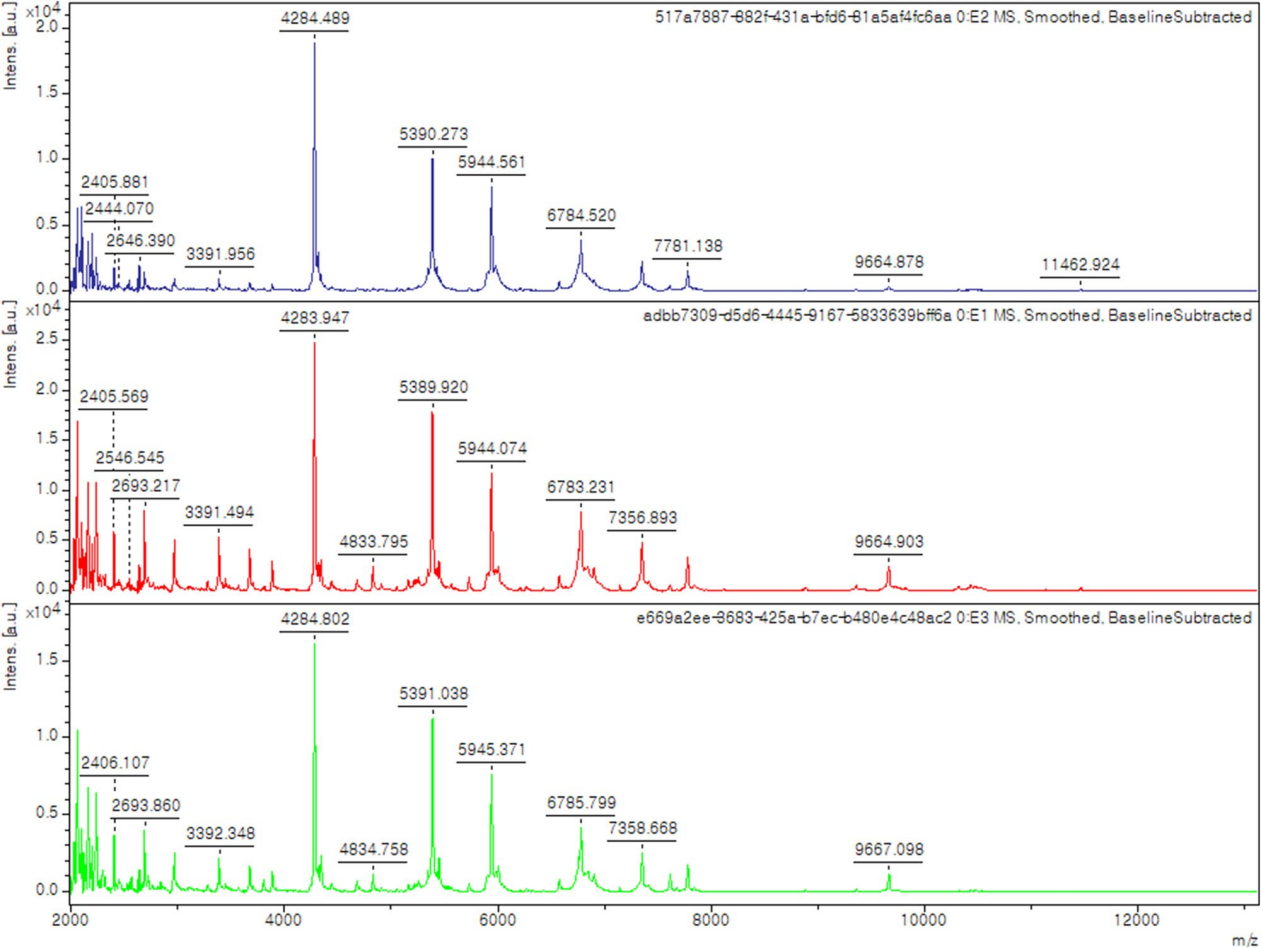
MALDI-TOF MS spectra of *Selenobaculum gbiensis* strain ICN-92133^T^.

**Figure 2:**
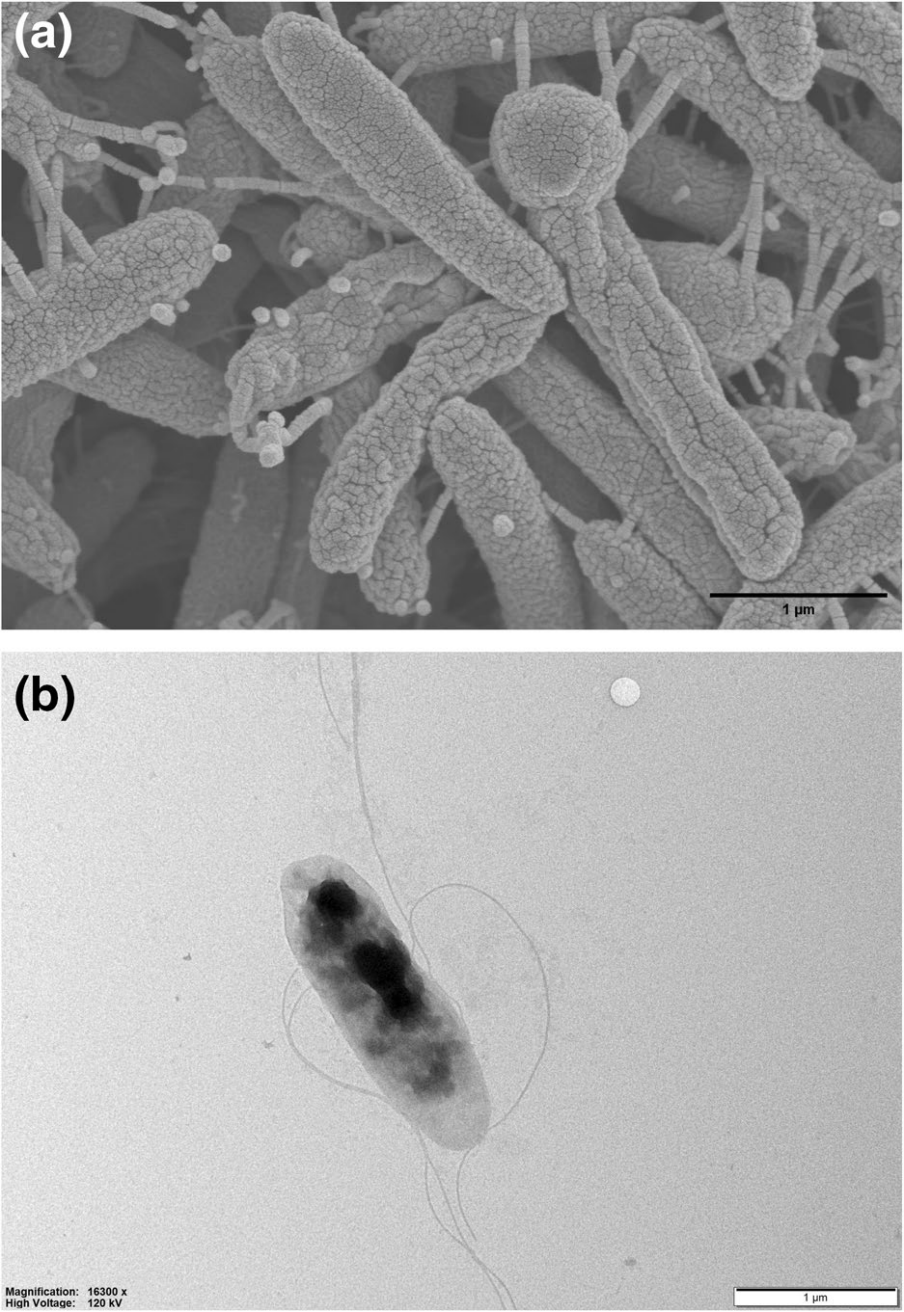
Morphology of *Selenobaculum gbiensis* strain ICN-92133^T^. Images were obtained using (**a**) scanning electron microscope (SEM) and (**b**) energy-filtered transmission electron microscope (EFTEM). Scale bars of 1 μm were represented.

**Figure 3:**
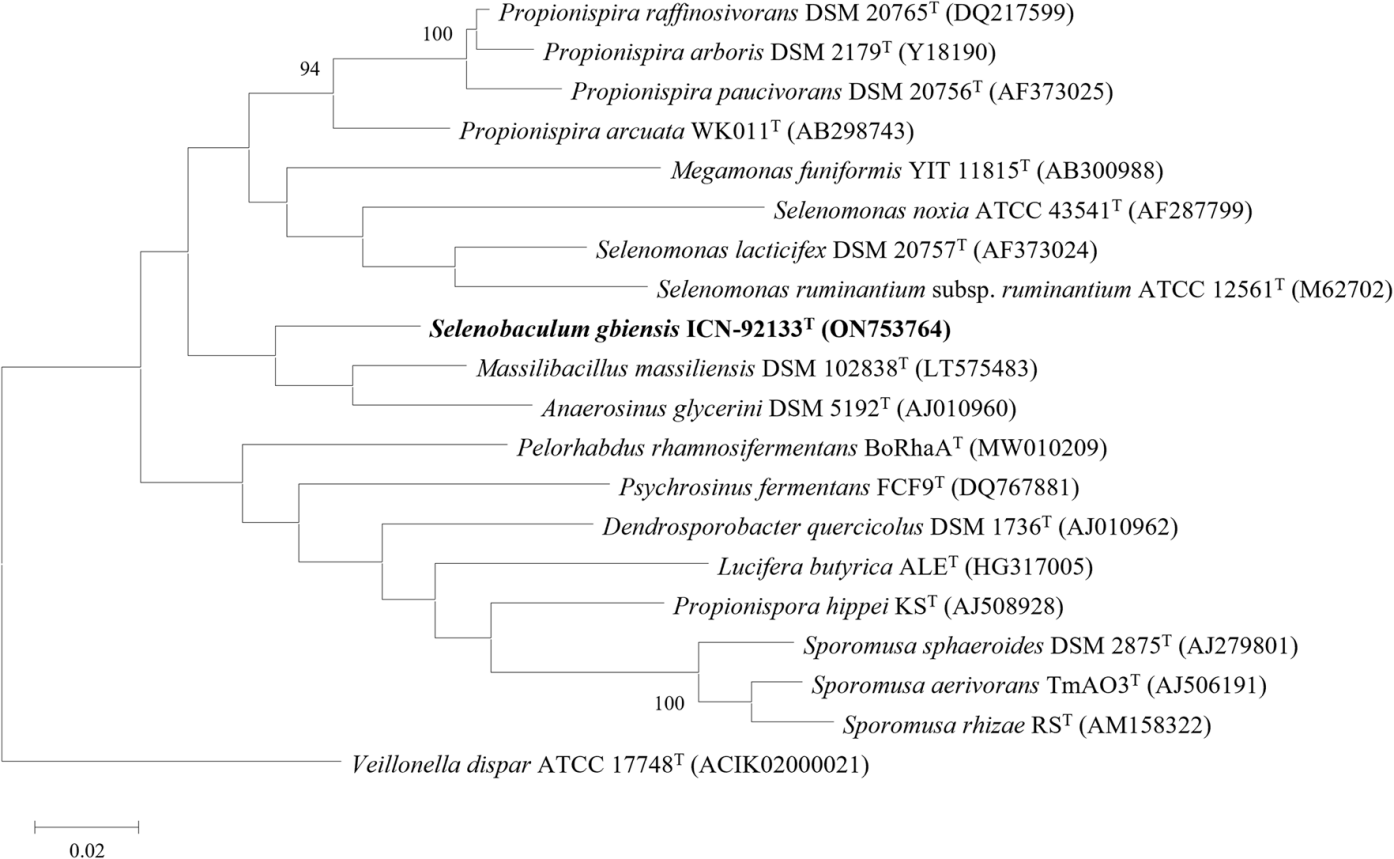
Phylogeny of *Selenobaculum gbiensis* strain ICN-92133^T^ and closely related species. The 16s rRNA sequences were aligned using MUSCLE with default parameters, and the phylogenetic tree was calculated using the maximum-likelihood method within MEGA software version 11.0. GenBank sequence accession numbers were described in parentheses. Among the bootstrap values obtained from 500 replications, values higher than 90% were indicated at branch points. *Veillonella dispar* was used as outgroup.

A colony of strain ICN-92133^T^ was first isolated on mGAM agar plates after 21 days of pre-incubation. The morphology of the colonies was creamy white, convex, and circular forms less than 1 mm in diameter. The spectrum of strain ICN-92133^T^ did not match the bacterial spectra in the MALDI Biotyper database showing scores lower than 1.5 (Fig. 1). The strain was gram-negative rod-shaped with 0.4–0.5 × 1.6–3.2 μm in size. However, some cells had blunt ends sporadically, which were observed in surface observations (Fig. 2a). In addition, structures presumed to be granules were observed inside the cells (Fig. 2b). The 16s rRNA gene sequence of strain ICN-92133^T^ was 1557 bp in length. Strain ICN-92133^T^ exhibited sequence similarity with *M. massiliensis* strain DSM 102838^T^ (93.91%)^37^, *Propionispira raffinosivorans* strain DSM 20765^T^ (93.08%)^38^, *Propionispira arcuata* strain KCTC 15499^T^ (92.73%)^39^, *Propionispira arboris* strain DSM 2179^T^ (92.51%)^40^, *Anaerosinus glycerini* strain DSM 5192^T^ (92.54%)^41,42^, and *Propionispira paucivorans* strain DSM 20756^T^ (91.85%)^38^. Phylogenetic analysis based on 16s rRNA placed strain ICN-91133^T^ into a member of the family *Selenomonadaceae* (Fig. 3). Since the highest identity value is lower than the threshold of 95% for determining a new genus, strain ICN-92133^T^ showed the possibility of being classified as a new genus of a new species and required further characterization.

### Comparison of phenotypic and biochemical characteristics

The condition from which strain ICN-92133^T^ was isolated was mGAM medium at 37 °C in an anaerobic atmosphere. Strain ICN-92133^T^ is motile with peritrichous flagella, but oxidase, nitrate reduction, and sporulation activities were not observed under the growth conditions (Table 1). The optimal growth conditions for strain ICN-92133^T^ were 26 to 37 °C, pH 6.5 to 8.5, and less than 0.5% salinity. To compare the main characteristics with strain ICN-92133^T^, four strains belonging to the genera *Massilibacillus*, *Propionispira,* and *Anaerosinus* were selected according to phylogeny results. Strain ICN-92133^T^ had enzymatic activities regarding pyroglutamyl aminopeptidase, alkaline phosphatase, esterase C4, esterase lipase C8, leucine arylamidase, acid phosphatase, and naphtol-AS-BI-phosphohydrolase. Moreover, the strain utilized glycerol, d-ribose, d-adonitol, d-glucose, d-fructose, d-mannose, and d-mannitol. Since *A. glycerini* strain DSM 5192^T^ utilizes glycerol as a primary carbon source, some reactions were not observed under the test conditions. In cellular fatty acids analysis, it is revealed that the primary cellular fatty acids of strain ICN-92133^T^ were C_17:1,*cis*-9_ (17.6%) and C_18:1,*cis*-9_ (19.5%) (Table 2).

**Table 1:** Biochemical characteristics of strain ICN-92133^T^ and its phylogenomically related type strains.

Properties	1	2	3	4	5
Isolation source	Stool from a patient with Crohn’s disease	Stool from a healthy infant	Methanogen reactor	Alkaline wet woods of poplar trees	Black freshwater sediment
Cell morphology	Rods with a rounded end	Rods with a rounded end	Curved rods	Curved rods with a rounded end	Curved rods
Cell size (μm)	0.4–0.5 × 1.6–3.2	0.4–0.6 × 1.7–3.5	0.3–0.5 × 1.3–2.7	0.6–0.9 × 3.1–7.2	0.3–0.6 × 2.3–4.1
Gram-staining	−	− (+)	−	−	−
Catalase activity	−	−	−	−	−
Oxidase activity	−	−	−	−	−
Nitrate reduction activity	−	−	−	−	−
Sporulation ability	−	−	−	−	−
Optimal growth conditions					
Temperature (20–45 °C)	26–37	26–37	20–30	20–30	26–37
pH (5.0–8.5)	6.5–8.5	6.5–8.5	6.5–7.5	6.0–7.0	6.5–8.5
NaCl (0–20 g/L)	< 5 g/L	< 15 g/L	< 3 g/L	< 3 g/L	< 3 g/L
Enzymatic activity					
Pyroglutamyl aminopeptidase	+	+	+	+	+
Alkaline phosphatase	+	+	+	+	−
Esterase (C4)	+	+	+	+	+
Esterase lipase (C8)	+	−	+	−	−
Leucine arylamidase	+	+	+	+	−
Acid phosphatase	+	+	+	+	+
Naphtol-AS-BI-phosphohydrolase	+	+	+	+	+
α-Galactosidase	−	−	−	+	−
β-Galactosidase	−	−	−	+	−
α-Glucosidase	−	−	−	+	−
β-Glucosidase	−	+	−	+	−
*N*-Acetyl-β-glucosaminidase	−	−	+	+	−
Substrate utilization					
Glycerol	+	−	+	+	+
Erythritol	−	−	+	+	NA
d-Arabinose	−	+	−	+	NA (−)
l-Arabinose	−	+	−	+	NA (−)
d-Ribose	+	+	+	+	NA (−)

Properties	1	2	3	4	5
d-Xylose	−	+	−	+	NA (−)
d-Adonitol	+	+	+	−	NA
d-Galactose	−	−	+	+	NA
d-Glucose	+	+	+	+	NA (−)
d-Fructose	+	+	+	+	NA
d-Mannose	+	+	+	+	NA (−)
l-Sorbose	−	−	−	+	NA (−)
l-Rhamnose	−	−	−	+	NA
Dulcitol	−	+	−	+	NA
Inositol	−	+	−	+	NA
d-Mannitol	+	+	+	+	NA (−)
d-Sorbitol	−	−	−	+	NA
Methyl-αd-Glucopyranoside	−	−	−	+	NA
*N*-Acetyl-glucosamine	−	+	−	+	NA
Amygdalin	−	+	−	+	NA
Arbutin	−	+	−	+	NA
Esculin, ferric citrate	−	+	−	+	NA
Salicin	−	+	−	+	NA (−)
d-Cellobiose	−	+	−	+	NA
d-Maltose	−	−	−	+ ( −)	NA (−)
d-Lactose (bovine origin)	−	−	−	+	NA (−)
d-Melibiose	−	−	−	+ ( −)	NA (−)
d-Saccharose (sucrose)	−	−	+	+	NA (−)
d-Trehalose	−	−	−	+	NA (−)
d-Raffinose	−	−	−	+	NA (−)
Amidon (starch)	−	−	−	+	NA
Gentiobiose	−	+	−	+	NA
d-Turanose	−	−	−	+	NA
d-Tagatose	−	+	+	+	NA
l-fucose	−	−	−	+	NA
d-Arabitol	−	+	−	+	NA
l-Arabitol	−	+	+	−	NA
Potassium gluconate	−	+	−	+	NA
Potassium 5-ketogluconate	−	+	−	−	NA

Strains: 1, ICN-92133^T^; 2, *Massilibacillus massiliensis* DSM 102838^T^; 3, *Propionispira arcuate* KCTC 15499^T^; 4, *Propionispira arboris* DSM 2179^T^; 5, *Anaerosinus glycerini* DSM 5192^T^.

Data different from the original paper are indicated in parentheses.

*NA* not available.

**Table 2:** Cellular fatty acids compositions (%).

Cellular fatty acids	1	2	3	4	5
C9:0	2.0	–	0.7	–	0.8
C10:0 iso	0.6	–	–	–	2.1
C10:0	2.4	–	1.1	–	3.4
C11:0	6.1	7.9	7.5	8.6	1.9
C13:0	0.3	–	–	2.6	–
C12:0 3-OH	3.9	3.6	0.7	–	8.3
C14:0 DMA	9.4	13.2	10.0	9.4	3.0
C15:1 Cis 7	8.3	10.8	14.7	27.3	3.3
C15:0	8.6	8.8	12.5	12.6	1.8
C15:0 DMA	–	–	1.4	3.3	–
C16:1 Cis 7	1.6	–	2.8	2.6	2.1
C16:0	3.3	3.6	2.3	1.3	6.0
C17:1 Cis 8	–	–	–	3.3	–
C17:1 Cis 9	17.6	18.5	23.3	5.7	2.0
C17:0	8.1	3.1	5.1	1.7	0.5
UN 17.223	–	–	1.2	3.6	–
C18:1 AT 17.254 DMA	–	–	4.3	5.1	–
C18:2 Cis 9,12	0.7	–	–	–	2.3
C18:1 Cis 9	19.5	30.7	10.3	9.5	53.6
C18:1c11/t9/t6	1.0	–	–	–	2.7

Strains: 1, ICN-92133^T^; 2, *Massilibacillus massiliensis* DSM 102838^T^; 3, *Propionispira arcuate* KCTC 15499^T^; 4, *Propionispira arboris* DSM 2179^T^; 5, *Anaerosinus glycerini* DSM 5192^T^.

The values above 2% in at least one strain are indicated. –, not detected.

### Antibiotic susceptibility of strain ICN-92133^T^

Strain ICN-92133^T^ showed MICs (μg/mL) of 0.094 for ampicillin, 4 for gentamicin, 4 for kanamycin, 6 for streptomycin, 6 for erythromycin, 0.032 for clindamycin, 0.19 for tetracycline, 0.5 for chloramphenicol, and more than 256 for vancomycin.

### Genomic properties of strain ICN-92133^T^

**Figure 4:**
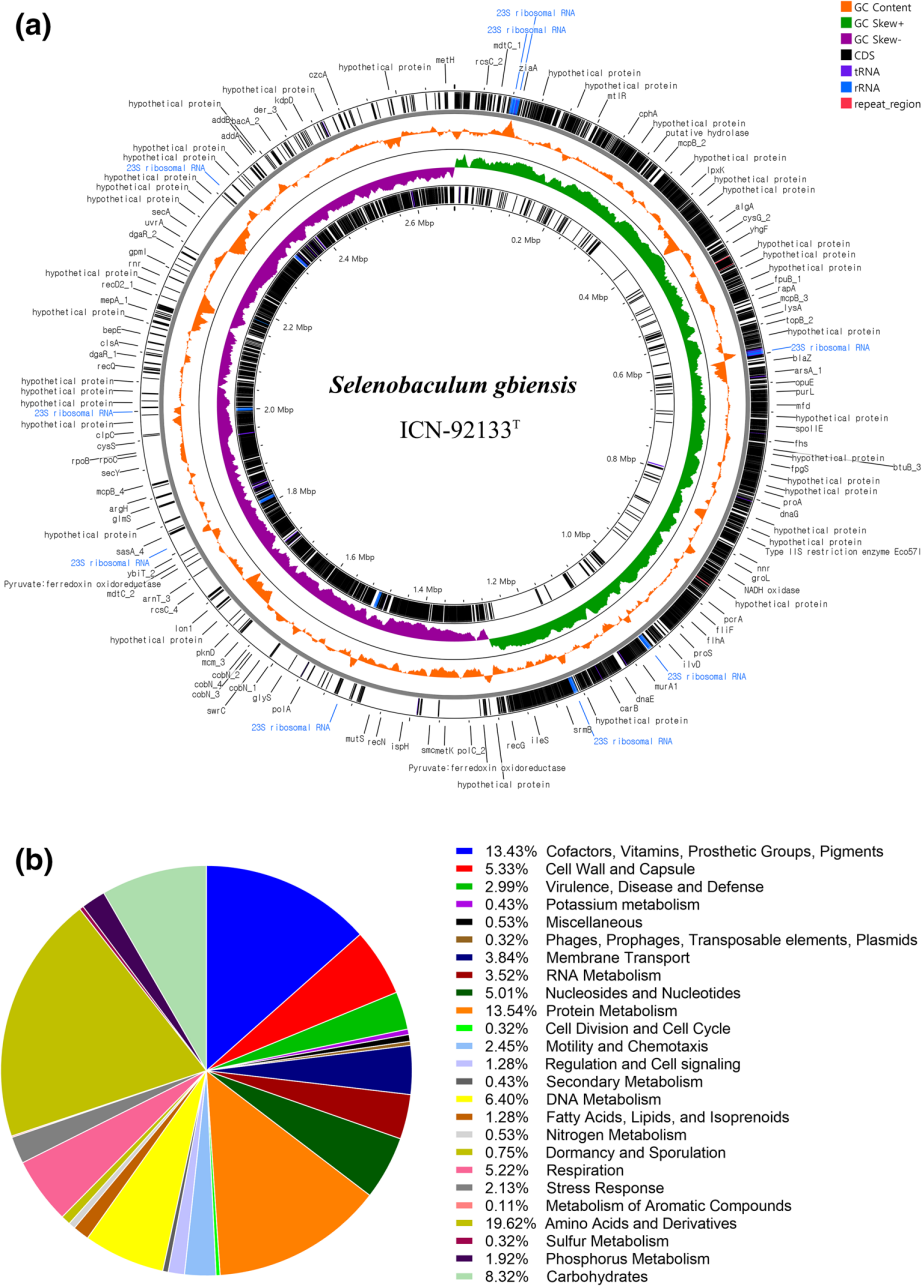
Complete genome and RAST subsystem category distributions of *Selenobaculum gbiensis* strain ICN-92133^T^. (**a**) A circular chromosome map represents annotated proteins, GC contents, forward GC skew, reverse GC skew, CDS, tRNA, rRNA, and repeat regions from outside to the center. (**b**) COG categories distributions were analyzed in the RAST server.

The complete genome of strain ICN-92133^T^ was a circular genome with a 2,679,003 bp size and a GC content of 35.5% (Fig. 4a). The genome contained 2526 protein-coding sequences (CDS), 86 tRNA genes, and 18 rRNA genes. The annotated proteins included 1016 hypothetical proteins and 1510 proteins assigned to 237 RAST subsystems (Fig. 4b). The clusters of orthologous group (COG) functional categories in which most of the genes involved were amino acids and derivatives (19.6%), protein metabolism (13.5%), cofactors, vitamins, prosthetic groups, pigments (13.4%), carbohydrates (8.3%) and DNA metabolism (6.4%). In addition, virulence factor candidates and antibiotic-resistance genes were predicted within the genome of strain ICN-92133^T^ to evaluate the potential risk to the host. A total of 38 sequences were matched to virulence genes in the VFDB database regarding adherence (*pilA*, *tcpl*, *groEL*, *tufA*, and *htpB*), exotoxin (*hlyB*), chemotaxis (*acfB*), immune modulation (*cpsB/cdsA*, *pseC*, *rfaD*, *gmd*, and *manC*), motility (*flhA*, *tlpB*, *gleQ*, and *pseB*), stress survival (*clpE* and *clpC*), and Type III secretion system (T3SS; *ysaV*, *escV,* and *invA*) (Table 3). In addition, five genes related to antibiotic efflux pump (*adeF* and *qacJ*), antibiotic target alteration (*vanT* and *vanW*), and inactivation of antibiotics (*fos*^*XCC*^) were detected in CARD.

**Table 3:** Prediction of virulence factors and antimicrobial resistance genes in the genome of *Selenobaculum gbiensis* strain ICN-92133^T^ based on VFDB and CARD databases, respectively.

Gene	Protein	Identity%	Database ID
Virulence factor database (VFDB)			
*tufA*	Elongation factor Tu	80.3/81.9/88.7^a^	VFG046465
*htpB*	Hsp60, 60 K heat shock protein HtpB	82.2/90.6/92.3^a^	VFG001855
*groEL*	Chaperonin GroEL	83.7/86.4^b^	VFG012095
*clpC*	Endopeptidase Clp ATP-binding chain C	88.1/90.4/95.7^a^	VFG000079
*escV*	Type III secretion system major export apparatus protein	84.9	VFG033545
*escV*	Type III secretion system major export apparatus protein	84.9	VFG000813
*clpE*	ATP-dependent protease	86.5/89.1/94.3^a^	VFG000080
*pseC*	UDP-4-amino-4, 6-dideoxy-*N*-acetyl-beta-l-altrosamine transaminase	83.7	VFG037927
*invA*	Type III secretion system major export apparatus protein InvA	82.7	VFG000557
*hlyB*	Hemolysin B	88.3/89.1^b^	VFG000841
*pseB*	UDP-GlcNAc-specific C4,6 dehydratase/C5 epimerase	84.5/88.5^b^	VFG012002
*gmd*	GDP-mannose 4,6-dehydratase	83.9	VFG002225
*fleQ*	Transcriptional regulator FleQ	94.74	VFG043319
*gmd*	GDP-mannose 4,6-dehydratase	79.75	VFG048885
*tlpB*	Membrane-bound chemoreceptor sensing pH and autoinducer-2	83.95	VFG043367
*pseB*	UDP-*N*-acetylglucosamine 4,6-dehydratase	92.68	VFG037912
*pilA*	Type IV major pilin protein PilA	94.44	VFG006896
*acfB*	Accessory colonization factor AcfB	83.75	VFG000104
*gmd*	GDP-mannose 4,6-dehydratase	79.35	VFG002365
*flhA*	Flagellar biosynthesis protein	90.7	VFG006577
*rfaD*	ADP-l-glycero-d-mannoheptose-6-epimerase	89.36	VFG000332
*ysaV*	EscV/YscV/HrcV family type III secretion system export apparatus	81.37	VFG003525
*tcpI*	Negative regulator of the major pilin TcpA	85.48	VFG000088
*manC*	Mannose-1-phosphate guanylyltransferase	92.11	VFG047006
*hlyB*	Hemolysin B	87.0/92.1^b^	VFG000907
*cpsB/cdsA*	Phosphatidate cytidylyltransferase	92.1	VFG002189
Comprehensive antibiotic resistance database (CARD)			
*adeF*	Resistance-nodulation-cell division (RND) antibiotic efflux pump	42.7	NA
*qacJ*	Small multidrug resistance (SMR) antibiotic efflux pump	47.6	NA
*vanT*	Glycopeptide resistance gene cluster	37.7	NA
*vanW*	Vancomycin B-type resistance protein VanW	28.1	NA
*fos*^XCC^	Fosfomycin thiol transferase	62.4	NA

*NA* not available.

^a^Three sequences were detected in the VFDB database. Each percentage of identity is represented.

^b^Two sequences were detected in the VFDB database. Each percentage of identity is represented.

### Comparative genomic characteristics

**Figure 5:**
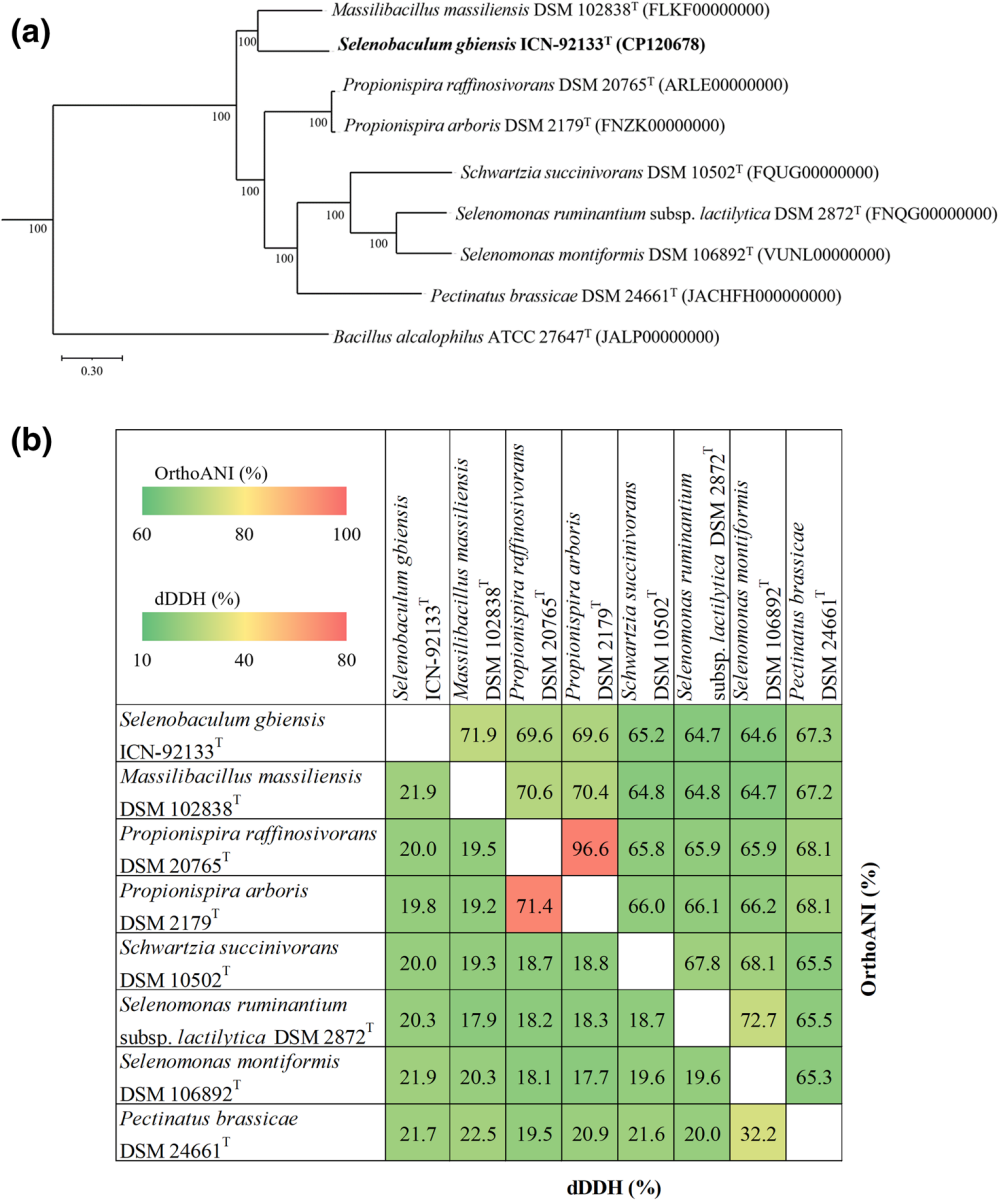
Whole genome-based phylogeny and pairwise comparison. (**a**) Phylogenetic tree based on Codon Tree pipeline in BV-BRC PGFams. GenBank sequence accession numbers were described in parentheses. *Bacillus alcalophilus* was used as outgroup. (**b**) Comparison of ANI and dDDH values between *Selenobaculum gbiensis* strain ICN-92133^T^ and closely related species. ANI values were obtained from OAT software, and dDDH was calculated in a formula 2 equation (DDH estimates based on identities/high-scoring segment pair length) in GGDC.

The whole genome of strain ICN-92133^T^ was compared with the available genomes of seven strains which were phylogenetic neighbors (Table 4). The genome of strain ICN-92133^T^ had the third shortest genome length and the lowest GC content and CDS numbers. In addition, strain ICN-92133^T^ showed a G+C content difference of 2.23 to 17.21% from the type strains excluding *Pectinatus brassicae* strain DSM 24661^T^. The subsystems annotated using the RASTtk are described in Table 5. The phylogenetic tree-based whole genomes constructed in PGfams Codon Tree pipeline showed that strain ICN-92133^T^ formed a clade with *M. massiliensis* strain DSM 102838^T^ and *Propionispira* type strains (Fig. 5a). Strain ICN-92133^T^ exhibited dDDH values lower than 21.9% and OrthoANI values ranging from 71.9% with *M. massiliensis* strain DSM 102838^T^ to 64.6% with *S. montiformis* strain DSM 106892^T^ (Fig. 5b). The dDDH and OrthoANI values were lower than the thresholds (70% and 95%, respectively), indicating that strain ICN-92133^T^ represented a novel species^43,44^. Taken together, we propose strain ICN-92133^T^ as a type species of a new genus within the family *Selenomonadaceae*.

**Table 4:** General genomic features of *Selenobaculum gbiensis* ICN-92133^T^ and related species.

Strains	GenBank accession number	Contigs	Genome size (bp)	CDS	G+C (mol, %)	Diff. G+C (%)^a^
*Selenobaculum gbiensis* ICN-92133^T^	CP120678	1	2,679,003	2526	35	0
*Massilibacillus massiliensis* DSM 102838^T^	FLKF00000000	7	4,000,953	3876	38	2.23
*Propionispira raffinosivorans* DSM 20765^T^	ARLE00000000	66	4,131,539	3945	38	2.65
*Propionispira arboris* DSM 2179^T^	FNZK00000000	40	4,261,607	4164	38	2.53
*Schwartzia succinivorans* DSM 10502^T^	FQUG00000000	21	2,659,575	2556	47	11.35
*Selenomonas ruminantium* subsp. *lactilytica* DSM 2872^T^	FNQG00000000	22	3,039,002	2950	50	14.06
*Selenomonas montiformis* DSM 106892^T^	VUNL00000000	26	2,640,693	2686	53	17.21
*Pectinatus brassicae* DSM 24661^T^	JACHFH000000000	90	2,849,197	2824	36	0.46

Strains were selected based on TYGS analysis, and the genomic data was retrieved from the BV-BRC server.

^a^The G+C content difference (%) from the sequence of *Selenobaculum gbiensis* strain ICN-92133^T^.

**Table 5:** Comparison of subsystems based on PATRIC annotation results.

Subsystems	ICN-92133^T^	DSM 102838^T^	DSM 20765^T^	DSM 2179^T^	DSM 10502^T^	DSM 2872^T^	DSM 106892^T^	DSM 24661^T^
Metabolism	378	515	1056	1103	414	786	419	407
Protein processing	149	210	414	420	199	402	200	195
Energy	79	164	328	329	62	186	90	94
Cellular processes	88	85	148	160	61	150	80	68
DNA processing	57	78	128	155	60	124	76	64
Stress response, defense, virulence	81	66	103	100	43	90	60	57
RNA processing	51	47	93	93	40	89	48	43
Membrane transport	18	26	77	89	21	86	25	22
Cell envelope	21	25	50	47	14	46	17	20
Miscellaneous	4	6	14	12	6	10	6	7
Regulation and cell signaling	4	4	12	12	3	8	3	4

Strains: ICN-92133^T^, *Selenobaculum gbiensis*; DSM 102838^T^, *Massilibacillus massiliensis*; DSM 20765^T^, *Propionispira raffinosivorans*; DSM 2179^T^, *Propionispira arboris*; DSM 10502^T^, *Schwartzia succinivorans*; DSM 2872^T^, *Selenomonas ruminantium* subsp. *lactilytica*; DSM 106892^T^, *Selenomonas montiformis*; DSM 24661^T^, *Pectinatus brassicae.*

## Discussion

Since gut microbiome and its association with host health and disease have been focused on, intensive efforts are being made to cultivate the gut microorganisms to elucidate microbe-microbe and microbe-host interactions^9,45–47^. The culturomics approach can contribute to acquiring diverse gut microbes, including new species that have never been cultured^8–12^. We obtained novel strains from the gut microbiota of healthy individuals and IBD patients during the culturomics study. Among them, the characteristics of strain ICN-92133^T^ are presented here with the taxonogenomic strategy.

The family *Selenomonadaceae*, which has the characteristics of strictly anaerobic and gram-negative bacilli, was first classified as a new family within the order *Selenomonadales* in the class *Negativicutes* in 2015^48^. The family currently contains 43 species in 8 genera, including *Anaerovibrio*, *Centipeda*, *Megamonas*, *Mitsuokella*, *Pectinatus*, *Propionispira*, *Schwartzia*, and *Selenomonas*. The phylogenetic analysis based on 16s rRNA gene sequences placed strain ICN-92133^T^ with species belonging to the family *Selenomonadaceae*, *Veillonellaceae,* and *Sporomusaceae* (Fig. 3). Phylogeny based on both amino acids and nucleotide sequences in the Codon Tree pipeline was inferred with currently available genomes within the three families to confirm its taxonomic classification (see Supplementary Fig. S1). Although strain ICN-92133^T^ not only showed the highest sequences similarity of 93.91% in 16s rRNA with *M. massiliensis* strain Marseille-P2411^T^ (= DSM 102838^T^), which belongs to family *Veillonellaceae*, but also constituted a clade in whole genome-based phylogeny, it shared a common ancestor with the other species of the family *Selenomonadaceae*. Therefore, we classified strain ICN-92133^T^ as a new genus belonging to the family *Selenomonadaceae*.

The complete genome of strain ICN-92133^T^ was smaller than those of *M. massiliensis* DSM 102838^T^ and *P. arboris* DSM 2179^T^, which could be the basis for the relatively fewer ranges of enzymatic and fermentation activities (Tables 1, 4). In electron microscopic observations of strain ICN-92133^T^, we found granular cytoplasmic inclusions in strain ICN-92133^T^ (Fig. 2b). Although the contained substances have not been verified in this study, they can be assumed to be a kind of energy storage based on the results of studies that *P. arboris* DSM 2179^T^ has carbohydrate-containing granules^40^. In addition, we investigated potential virulence and antibiotic resistance genes within the complete genome of strain ICN-92133^T^ to predict its pathogenicity because the strain was isolated from fecal specimens of a patient with Crohn’s disease (Table 4). Based on the strain ICN-92133^T^ results showing high MIC values for vancomycin in vitro, the resistance might be derived from efflux pumps and reduced binding affinity to vancomycin. Our results also included several housekeeping proteins in the VFDB results, such as EF-Tu and GLoEL, which may play a role in the moonlighting functions in certain species^49,50^. However, it is necessary to confirm whether the detected genes in strain ICN-92133^T^ are expressed in the host and show pathogenicity through further research.

In conclusion, we propose strain ICN-92133^T^ obtained through culturomics as a type species *Selenobaculum gbiensis* sp. nov., of a new genus *Selenobaculum* within the family *Selenomonadaceae*, according to the taxonogenomic results, including MALDI-TOF MS spectra, morphology, phenotypic properties, phylogenetic analysis, FAME composition, ANI, and dDDH calculation.

### Description of *Selenobaculum* gen. nov.

*Selenobaculum* (Se.le.no.ba.cu.lum. N.L. fem. pl. n. *Selenomonadaceae*, a bacterial family name; L. neut. n. *baculum*, a stick, staff, rod; N.L. neut. n. *Selenobaculum*, a rod-shaped bacterium that belongs to the family *Selenomonadaceae*). Cells are gram-negative rods with rounded ends, obligately anaerobic, motile with flagellum, oxidase, and catalase negative. The type species is *Selenobaculum gbiensis*.

### Description of *Selenobaculum gbiensis* gen. nov., sp. nov.

*Selenobaculum gbiensis* (g.bi.en’sis. N.L. masc. adj. *gbiensis* for the acronym of Green-Bio Institute, where the type strain was isolated). Cells grow at 26–37 °C, pH 6.5–8.5, a salinity of less than 0.5%, and the size was 0.4–0.5 × 1.6–3.2 μm. After 2 days of incubation at 37 °C in anaerobic conditions, colonies that were creamy and circular forms less than 1 mm in diameter were observed on mGAM agar. Among the 19 enzymatic activities tested in the API ZYM system, a positive reaction was observed for pyroglutamyl aminopeptidase, alkaline phosphatase, esterase (C4), esterase lipase (C8), leucine arylamidase, acid phosphatase, and naphtol-AS-BI-phosphohydrolase. In API 20A and API 50CH tests, it was observed to be utilized glycerol, d-ribose, d-adonitol, d-glucose, d-fructose, d-mannose, and d-mannitol as carbon sources. In contrast, utilization for other substrates was not observed. The primary cellular fatty acids were C_17:1,*cis*-9_ and C_18:1,*cis*-9_. The type strain ICN-92133^T^ was first isolated from a patient’s stool with Crohn’s disease, and its genome was found to be 2,679,003 base-pair lengths and 35.5 mol% of GC contents. Strain ICN-92133^T^ has been deposited at Korean Collection for Type Cultures (KCTC) and the Japan Collection of Microorganisms (JCM) under the numbers KCTC 25622^T^ and JCM 36070^T^, respectively.

### Supplementary Information

Supplementary Figure S1.

## Data Availability

The 16s rRNA gene sequence and complete genome sequence for strain ICN-92133^T^ have been deposited under GenBank Accession No. ON753764 and CP120678 (BioProject accession no. PRJNA944274, BioSample accession no. SAMN33746654, and SRA accession no. SRR23852393–SRR23852394), respectively.
