# Supplementary material for: Selenobaculum gbiensis gen. nov. sp. nov., a new bacterium isolated from the gut microbiota of a patient with Crohn’s disease
Source: Sci Rep. 2023 Sep 8;13:14835. doi: 10.1038/s41598-023-42017-0 (PMC10491768; doi:10.1038/s41598-023-42017-0)
Supplement: Supplementary file 1 — Supplementary Figure S1. [file 41598_2023_42017_MOESM1_ESM.pdf]

Selenobaculum gbiensis gen. nov. sp. nov., a new bacterium isolated from the gut microbiota of a patient with Crohn's disease

Soyoung Yeo, Hyunjoon Park, Heebal Kim, Chang Beom Ryu and Chul Sung Huh

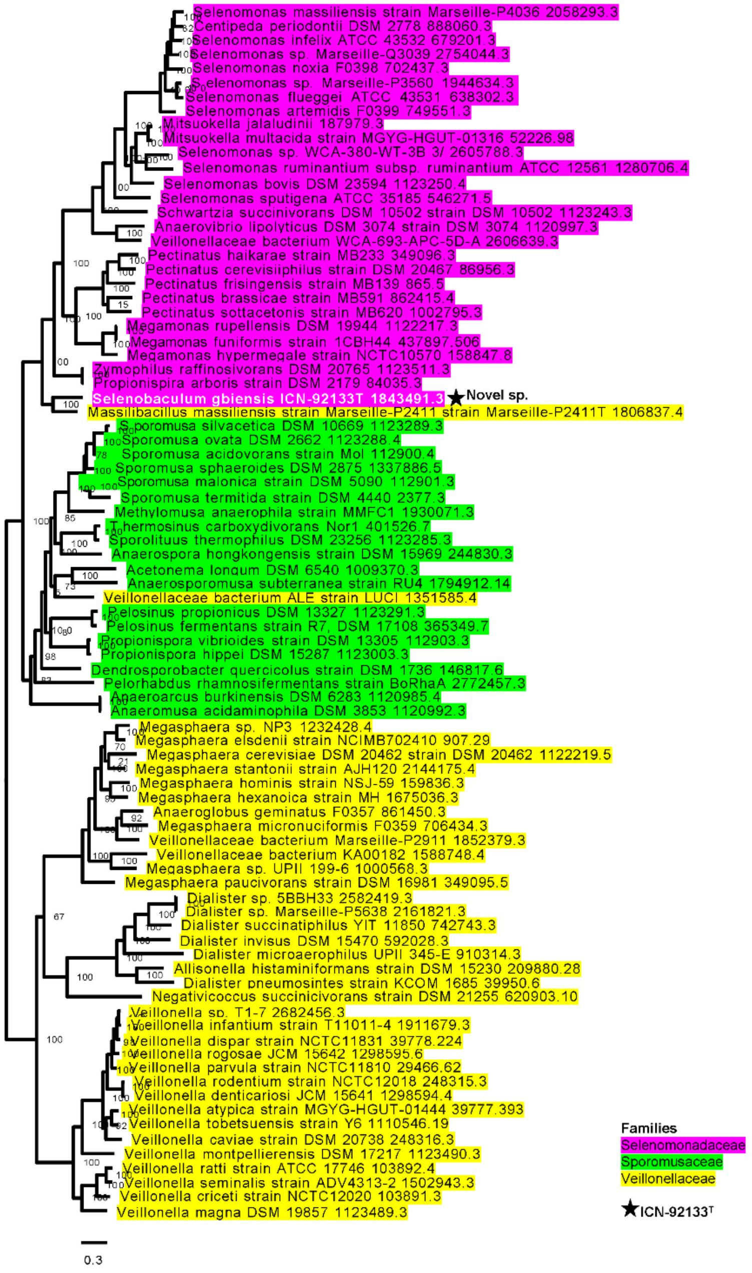

Figure S1. Whole-genome phylogeny of three families (*Selenomonadaceae*, *Sporomusaceae,* and *Veillonellaceae*) inferred by PGfams Codon Tree pipeline.
